# Machine learning‐assisted prediction of clinical responses to periodontal treatment

**DOI:** 10.1002/JPER.24-0737

**Published:** 2025-04-20

**Authors:** Balazs Feher, Eduardo H. de Souza Oliveira, Poliana Mendes Duarte, Andreas A. Werdich, William V. Giannobile, Magda Feres

**Affiliations:** ^1^ Department of Oral Medicine, Infection, and Immunity Harvard School of Dental Medicine Boston Massachusetts USA; ^2^ Department of Oral Surgery University Clinic of Dentistry, Medical University of Vienna Vienna Austria; ^3^ Department of Oral Biology University Clinic of Dentistry, Medical University of Vienna Vienna Austria; ^4^ Topic Group Dental Diagnostics and Digital Dentistry International Telecommunication Union–World Health Organization–World Intellectual Property Organization Global Initiative on Artificial Intelligence for Health Geneva Switzerland; ^5^ Department of Periodontology University of Florida Gainesville Florida USA; ^6^ Core for Computational Biomedicine, Department for Biomedical Informatics, Blavatnik Institute Harvard Medical School Boston Massachusetts USA

**Keywords:** artificial intelligence, machine learning, periodontics, predictive medicine, treatment outcome

## Abstract

**Background:**

Periodontitis is among the most prevalent chronic inflammatory conditions globally, and is associated with bone resorption, tooth loss, and systemic complications. While its treatment is largely standardized, individual outcomes vary, with some patients experiencing further disease progression despite adherence.

**Methods:**

We developed a machine learning (ML) approach to predict individual outcomes 1 year post‐treatment using retrospectively assessed baseline parameters. We trained a Random Forest model on 18 demographic, clinical, microbiological, and treatment‐related features of 414 patients from randomized clinical trials (RCTs) in South America. We subsequently performed internal testing, interpretability analysis, and external testing on a second dataset of 78 patients from previous RCTs in North America and Europe exhibiting less severe disease.

**Results:**

In internal testing, the ML model achieved an area under the receiver operator characteristics curve (AUROC) of 0.93, an area under the precision‐recall curve (AUPRC) of 0.90, an F_1_‐score of 0.82, and an out‐of‐bag score of 0.71. Relative importances were 0.42 for clinical, 0.33 for treatment‐related, 0.21 for microbiological, and 0.04 for demographic features. In external testing, the ML model achieved an AUROC of 0.76, an AUPRC of 0.69, and an F_1_‐score of 0.71.

**Conclusions:**

Our study indicates that an ML‐based approach can assist in predicting individual responses to periodontal treatment. Prospective validation is needed for clinical application.

**Plain language summary:**

Using comprehensive data from patients with periodontitis, an inflammatory condition of the tooth‐supporting tissues, a machine learning model was trained to predict how well patients might respond to different treatments after 1 year. The model was externally tested in patient populations from 2 continents different from the training dataset. The results suggest that with further research and refinement, this tool could eventually become a valuable asset in personalizing treatment plans for improved patient outcomes.

## 1 INTRODUCTION

Despite decades of clinical research, treatment protocols for periodontitis, an etiologically complex, multifactorial inflammatory disease of the tooth‐supporting specialized tissues,^1^ largely adhere to a one‐size‐fits‐all approach. Current protocols rely on mechanical debridement and non‐specific antibiotic regimens, while addressing a limited range of individual risk factors.^2^, ^3^ This standardized approach reduces inflammation but still results in 30–70% of patients failing to achieve clinical treatment endpoints.^4^ A further 25–50% of patients experience disease progression, including tooth loss, even with optimal oral hygiene and consistent professional maintenance care.^5^, ^6^, ^7^ Affecting more than a billion patients globally, periodontitis ranks among the most prevalent medical conditions.^8^ With a rising incidence,^9^, ^10^ an estimated global economic burden in the hundreds of billions of dollars,^11^ and precise treatment recommendations remaining elusive, periodontitis remains a major clinical and public health challenge,^12^ prompting an urgent call for personalized approaches.^13^, ^14^

Advancements in disease understanding and applied data science have created a timely opportunity to overcome longstanding healthcare limitations and advance precision medicine.^15^ Several medical disciplines have made meaningful progress toward stratified diagnostics and treatment,^16^, ^17^ and artificial intelligence (AI) is increasingly used to estimate treatment responses.^18^ Despite the growing recognition of the importance of oral diseases in the context of overall health,^19^ dentistry has largely remained reliant on universal treatment guidelines.^20^ The progressive use of AI in dentistry is primarily driven by deep learning‐based diagnostic modeling ^21^; neural networks leverage the vast number of radiographs in clinical dental practice.^22^ In contrast to the increasing clinical adoption of diagnostic models, AI‐enabled prognostic modeling, treatment planning, and non‐image data applications remain underdeveloped. Despite increasing research on applied machine learning (ML) in dentistry,^23^ prognostic models rarely transcend academic boundaries. Consequently, while previous work has emphasized the clinical and public health benefits of risk stratification and tailored preventive strategies,^24^, ^25^ periodontal treatment planning does not adequately address the profound inter‐individual differences in microbial signatures, clinical manifestations, risk factors, disease progression, or treatment responses.

Previous research has used prognostic ML models to estimate periodontal risk^26^ and disease progression.^27^ While more granular than the official classification scheme,^28^ these models still primarily incorporated basic demographic and clinical features, overlooking individual microbial signatures and the impact of treatment choice. Our previous work has demonstrated that ML models trained on microbiological data could distinguish between broad periodontal disease categories,^29^ even when these were merged in the current classification scheme.^28^ A similar approach later integrated clinical, immunological, and microbiological data for profiling and risk stratification of patients with peri‐implantitis, a similar disease affecting dental implants.^30^ In contrast, there is limited evidence on the prediction of individual treatment responses.^31^ Consequently, a genuinely personalized approach to periodontal treatment, grounded in robust prognostic modeling, remains elusive.

Specific inter‐individual differences in clinical disease manifestation and microbial profile, both measurable prior to periodontal treatment, may lead to varying responses to different treatment approaches. The interaction between patient phenotypes and treatment choices might in turn explain why some patients fail to achieve clinical endpoints despite strict adherence to treatment protocols. This challenge might be best addressed by classical ML algorithms, which could more effectively balance performance in complex data with explainability, a key factor in clinical application.^32^ Therefore, the aim of this study was to establish a prognostic ML approach for the prediction of individual periodontal treatment outcomes. We hypothesized that baseline patient features could predict individual periodontal treatment responses. To test this hypothesis, we retrospectively collated a multi‐center set of raw patient data from randomized controlled trials (RCTs) conducted across 3 different continents. We used these data to train and test a prognostic ML model for individual treatment outcomes. To minimize false positives resulting from early treatment effects followed by relapse, we focused on predicting individual outcomes 1 year post‐treatment. The model was trained and internally tested using data from South America, followed by external testing on data from North America and Europe.

## 2 METHODS

### 2.1 Study design and data collection

**FIGURE 1:**
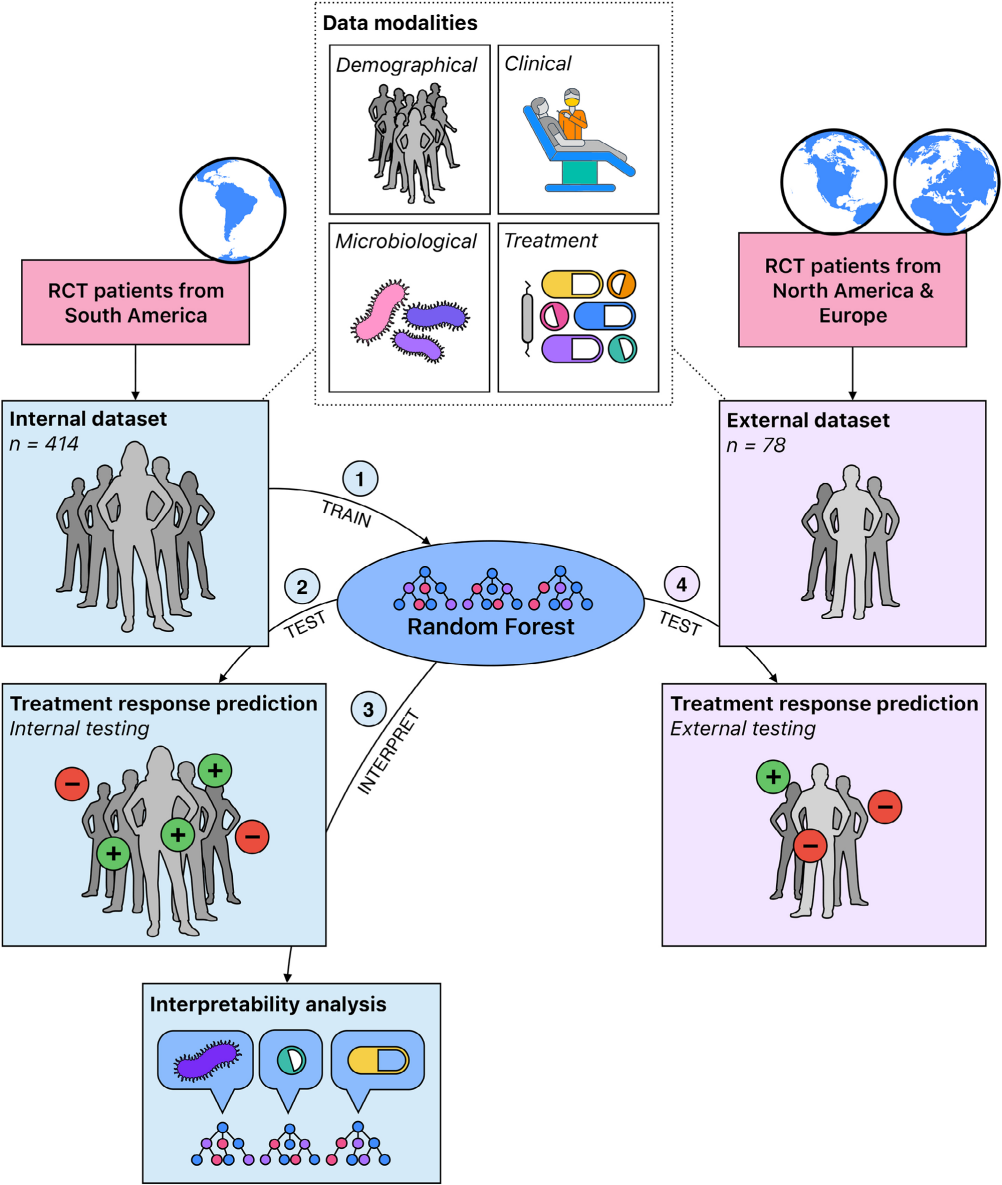
Overview of the study workflow. We collated a dataset for training and internal testing from the raw data of RCT patients from South America; we further collated a second dataset for external testing from raw RCT patient data from North America and Europe. Both datasets included demographical, clinical, microbiological, and treatment‐related parameters. First, we used the South American dataset to train a Random Forest model for the prediction of the previously validated clinical endpoint.^34^ We then performed internal testing as well as interpretability analysis of the model. Finally, we performed external testing of the model on the North American/European dataset. RCT, randomized controlled trial.

This retrospective study is based on a secondary analysis of raw RCT data. A high‐level overview of the study workflow is shown in Figure 1. The study design involved no patient or public involvement. No study protocol was prepared a priori. This study was not registered. All data were collected retrospectively. Reporting followed TRIPOD+AI guidelines.^33^

For training and internal testing, we collected raw data from 6 previous RCTs in South America (See Table S1 in online *Journal of Periodontology*).^35^, ^36^, ^37^, ^38^, ^39^, ^40^, ^41^, ^42^ Inclusion criteria were: (i) assessment of demographic, clinical, microbiological, and treatment‐related parameters; (ii) availability of clinical data at the site level (i.e., 6 measurements per tooth); (iii) availability of microbiological data from multiple sites per patient; (iv) availability of all data at baseline and 1 year post‐treatment. The South American dataset included 414 patients (median age: 45 years, interquartile range [IQR]: 37–53, 58% female; Table 1). For external testing, we collected raw data from 2 previous RCTs in North America and Europe^43^, ^44^, ^45^ (See Table S1 in online *Journal of Periodontology*). Data had to be fully compatible with the internal dataset, following the same structure and measurement criteria. The South American/European dataset included 78 patients (median age: 48 years, IQR: 39–57, 35% female; Table 1). The already de‐identified data were checked for missing values and possible errors before analysis. Data completeness was assessed at the tooth level. Patients with incomplete data (i.e., not all sites recorded) were excluded and only patients with complete data were included.

**TABLE 1:** Dataset characteristics.

Parameter	Internal dataset	External dataset
Source	South America	North America, Europe
Total number of patients	414	78
Age (median; IQR)	45 years; 37–53	48 years; 39–57
Sex (*n*; %)		
Male	175; 42%	51; 65%
Female	239; 58%	27; 35%
Clinical disease manifestation (mean ± SD)		
Sites with PPD ≥ 5 mm	42 ± 22	28 ± 21
Sites with CAL ≥ 5 mm	54 ± 24	32 ± 24
Percentage of sites with BoP	63% ± 22%	54% ± 27%
Percentage of sites with plaque	70% ± 20%	44% ± 27%
Microbiological load^46^ (mean ± SD)		
Red complex	65 ± 49	6 ± 5
Orange complex	98 ± 88	15 ± 12
Yellow complex	19 ± 22	2 ± 2
Green complex	22 ± 26	6 ± 8
Purple complex	18 ± 20	3 ± 2
Select *Actinomyces* species	30 ± 27	6 ± 5
Miscellaneous	33 ± 32	10 ± 16
Adjunctive treatment		
Antibiotics (*n*; %)	293; 71%	41; 53%
Antibiotic duration (mean ± SD)	9.9 ± 6.0 days	7.4 ± 7.0 days
Treatment outcome at 1 year (*n*; %)		
Endpoint achieved^34^	172; 42%	32; 41%
Endpoint not achieved^34^	242; 58%	46; 59%

*Note*: Red complex: *Tannerella forsythia, Porphyromonas gingivalis, Treponema denticola*. Orange complex: *Campylobacter gracilis, Campylobacter rectus, Campylobacter showae, Eubacterium nodatum, Fusobacterium nucleatum ssp. nucleatum, Fusobacterium nucleatum ssp. polymorphum, Fusobacterium nucleatum ssp. vincentii, Fusobacterium periodonticum, Parvimonas micra, Prevotella intermedia, Streptococcus constellatus, Prevotella nigrescens*. Yellow complex: *Streptococcus gordonii, Streptococcus intermedius, Streptococcus mitis, Streptococcus oralis, Streptococcus sanguinis*. Green complex: *Aggregatibacter actinomycetemcomitans, Capnocytophaga gingivalis, Capnocytophaga ochracea, Capnocytophaga sputigena, Eikenella corrodens*. Purple complex: *Actinomyces odontolyticus, Veillonella parvula*. Select Actinomyces species: *Actinomyces gerencseriae, Actinomyces israelii, Actinomyces naeslundii, Actinomyces oris, Actinomyces odondolyticus*. Miscellaneous: *Eubaterium saburreum, Gemella morbillorum, Leptotrichia buccalis, Propionibacterium acnes, Prevotella melaninogenica, Neisseria mucosa, Streptococcus anginosus, Selenomonas noxia, Treponema socranskii*.

Abbreviations: BoP, bleeding on probing; CAL, clinical attachment level; IQR, interquartile range; PPD, probing pocket depth; SD, standard deviation.

### 2.2 Feature engineering

The primary outcome parameter was the previously validated clinical endpoint of having at most 4 sites with probing pocket depth (PPD) ≥ 5 mm 1 year post‐treatment.^34^ This binary variable, calculated from site‐level PPD measurements, was the event of interest for the prediction model (decision threshold: 50%). After assessing its frequency in the dataset, we engineered predictive features to minimize loss of granularity while maintaining a low events‐per‐variable (EPV) ratio. PPD and clinical attachment loss (CAL) measurements were transformed from the site to the patient level by categorizing them according to thresholds; bleeding on probing (BoP) and plaque were transformed by calculating the percentage of positive sites. Demographic features included (i) age; and (ii) sex. Clinical features included (i) number of sites with PPD < 5 mm; (ii) number of sites with PPD ≥ 5 mm; (iii) number of sites with CAL < 5 mm; (iv) number of sites with CAL ≥ 5 mm; (v) percentage of sites with BoP; and (vi) percentage of sites with plaque. Microbiological variables included the total load of the following microbial complexes^46^: (i) red complex *(Tannerella forsythia, Porphyromonas gingivalis, Treponema denticola)*; (ii) orange complex *(Campylobacter gracilis, Campylobacter rectus, Campylobacter showae, Eubacterium nodatum, Fusobacterium nucleatum ssp. nucleatum, Fusobacterium nucleatum ssp. polymorphum, Fusobacterium nucleatum ssp. vincentii, Fusobacterium periodonticum, Parvimonas micra, Prevotella intermedia, Streptococcus constellatus, Prevotella nigrescens)*; (iii) yellow complex *(Streptococcus gordonii, Streptococcus intermedius, Streptococcus mitis, Streptococcus oralis, Streptococcus sanguinis)*; (iv) green complex *(Aggregatibacter actinomycetemcomitans, Capnocytophaga gingivalis, Capnocytophaga ochracea, Capnocytophaga sputigena, Eikenella corrodens)*; (v) purple complex *(Actinomyces odontolyticus, Veillonella parvula)*; (vi) select *Actinomyces* species *(Actinomyces gerencseriae, Actinomyces israelii, Actinomyces naeslundii, Actinomyces oris, Actinomyces odondolyticus)*; (vii) and total miscellaneous microbial load of *Eubaterium saburreum, Gemella morbillorum, Leptotrichia buccalis, Propionibacterium acnes, Prevotella melaninogenica, Neisseria mucosa, Streptococcus anginosus, Selenomonas noxia*, and *Treponema socranskii*. Treatment‐related features included (i) total daily amoxicillin dose; (ii) total daily metronidazole dose; (iii) antibiotic treatment duration. Feature assessment was not blinded during model development and testing.

### 2.3 Prognostic model training

To predict the clinical endpoint^34^ at the 1‐year follow‐up, a Random Forest model with a total of 18 demographic, clinical, microbiological, and treatment‐related input features was trained. A grid search was used for hyperparameter tuning. The final Random Forest model included 200 trees. Bootstrap sampling, and the out‐of‐bag method were used for internal validation. The maximum tree depth was set to 5. Each split considered 10 features. The trees were limited to 20 leaf nodes, with at least 8 samples per leaf; each internal node required at least 10 samples to split.

For benchmarking, we trained Extreme Gradient Boosting (XGBoost), Support Vector Machine, and K‐Nearest Neighbors models and compared their performance against our Random Forest model. Continuous variables in datasets used to train and test Support Vector Machine and K‐Nearest Neighbors models were normalized prior to model training. Grid searches were used for hyperparameter tuning (for a description of optimal hyperparameters, see Table S2 in the *online Journal of Periodontology)*.

We assessed standard performance metrics (e.g., area under the receiver operating characteristics curve [AUROC], area under the precision‐recall curve [AUPRC], out‐of‐bag score, F_1_‐score, calibration‐at‐large). We further performed calibration curve and decision curve analysis to evaluate clinical usefulness, comparing the model to the “all” and “none” (i.e., every or no patient achieves the endpoint, respectively) assumptions. Finally, we performed subgroup analysis based on the number of sites with CAL ≥ 5 mm. Model training and testing were performed in a Python environment (version 3.13.0) using the scikit‐learn library (version 1.5.2).

### 2.4 Interpretability analysis

We evaluated the relative importance of each input feature, highlighting which features the model relies on most heavily when making predictions. Relative feature importance is a quantitative measure of the extent to which individual features contribute to overall predictions. Representing their collective contribution to the model's prediction process, the sum of all relative feature importance's equals 1.

Additionally, we generated partial dependence plots, providing a graphical representation of how changes in each of the key input features affect the model's predictions under the assumption that all other features are held constant, thereby illustrating the marginal effect of individual features on the achievement of the clinical endpoint^34^ (e.g., the partial dependence plot for total metronidazole dosage shows how variations in dosage levels independently impact the probability of treatment success). Finally, we generated 2‐dimensional partial dependence plots showing the combined marginal effects of clinically relevant pairs of predicting features.

## 3 RESULTS

### 3.1 Prognostic performance in internal testing

**FIGURE 2:**
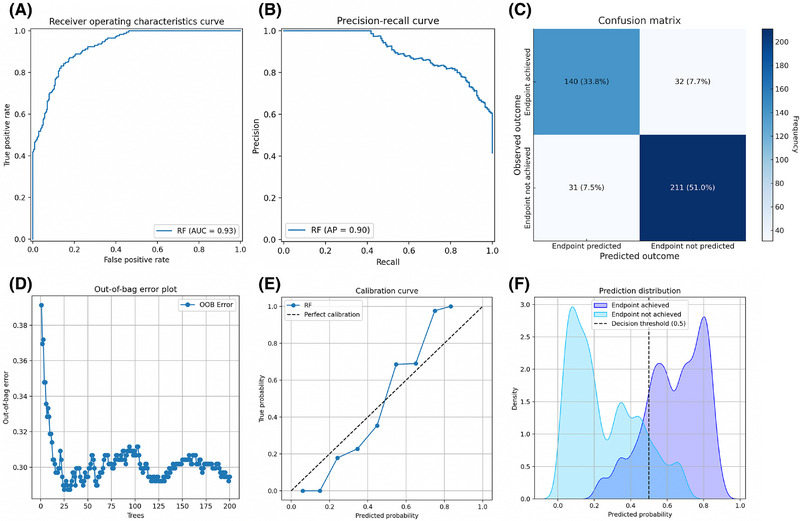
Prognostic model performance in internal testing. Performance is shown as evaluated on the South American dataset (*n* = 414) used for training. (A) Receiver operating characteristics curve. (B) Precision‐recall curve. The model achieved a precision of 0.82 and a recall of 0.81. (C) Confusion matrix. The model achieved an accuracy of 0.85. (D) Out‐of‐bag error plot. The out‐of‐bag score was 0.71, calculated using an average of 152 ± 7 out‐of‐bag samples per tree across 200 trees. (E) Calibration curve. (F) Prediction distribution. Densities are normalized. AP, average precision; AUC, area under the curve; RF, Random Forest.

In internal testing on the dataset of South American patients, our prognostic model predicted a mean probability of 42% of achieving the clinical endpoint,^34^ compared with an observed events fraction of 42%. The model achieved an AUROC of 0.93 (Figure 2A). The model's precision, recall, and AUPRC were 0.82, 0.81, and 0.90, respectively; the F_1_‐score was 0.82 (Figure 2B). The model achieved an accuracy of 0.85, with an error distribution of 0.08 for both false negatives and false positives (Figure 2C). The out‐of‐bag score was 0.71, calculated using an average of 152 ± 7 out‐of‐bag samples per tree across 200 trees (Figure 2D). The calibration curve had a slope of 1.45 (95% confidence interval: [1.23, 1.65]; Figure 2E). Distribution analysis showed a clear separation of predicted outcomes (Figure 2F). Decision curve analysis demonstrated a clear net benefit advantage across relevant threshold probabilities, including the 50% decision threshold (See Figure S1A in online *Journal of Periodontology*). Subgroup analysis based on the number of sites with CAL ≥ 5 mm showed consistent model performance across the first 3 quartiles (AUROC: 0.91–0.94; F_1_‐score: 0.83–0.84), with a decrease in performance in the fourth quartile (AUROC: 0.92; F_1_‐score: 0.65; see Table S3 in online *Journal of Periodontology*).

### 3.2 Model interpretability

**FIGURE 3:**
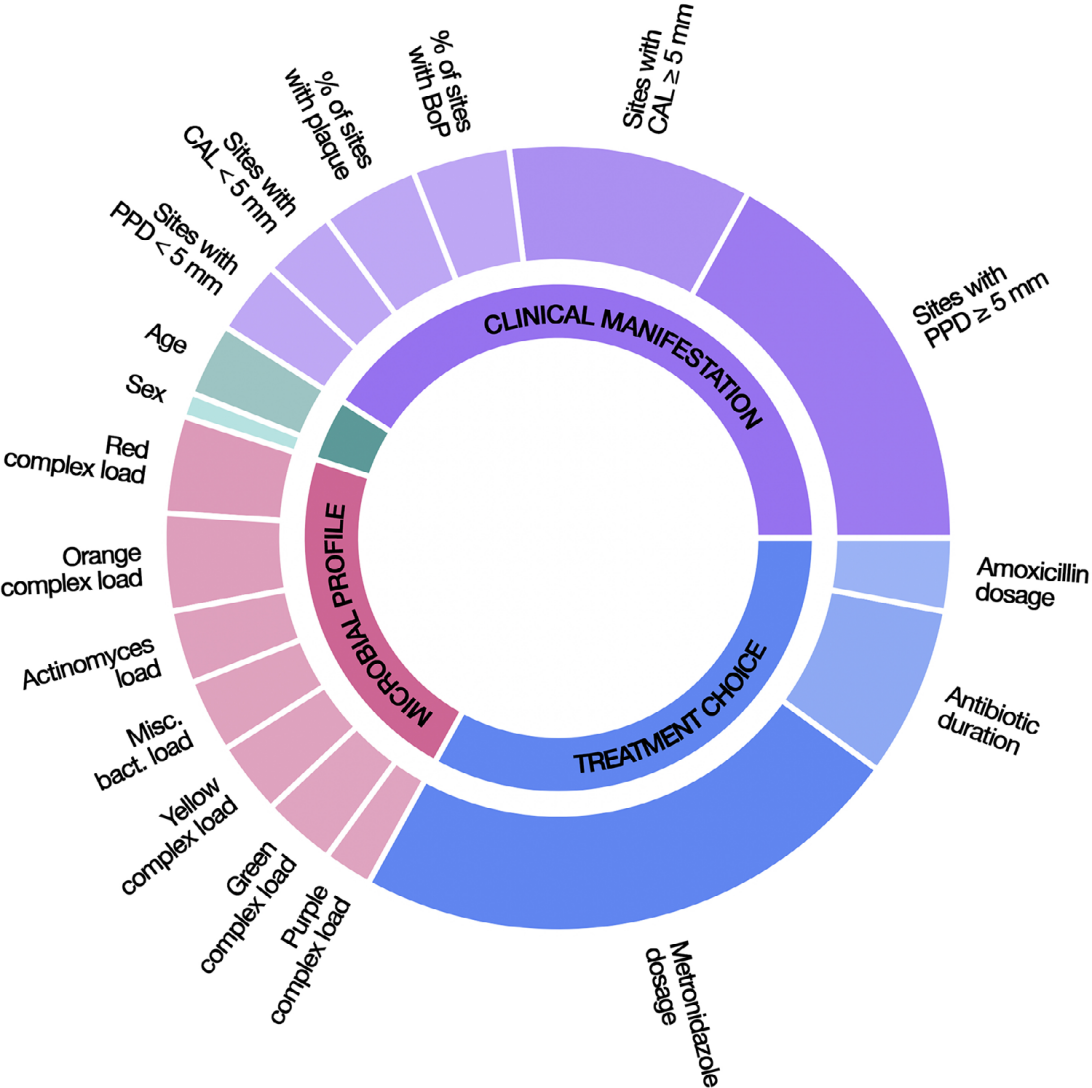
Relative feature importance's. The collective relative importance's of clinical (0.40), treatment‐related (0.30), and microbiological (0.26) features are comparable. Individually, the features with the highest relative importance's are total daily metronidazole dose (0.18), number of sites with a PPD of at least 5 mm (0.12), number of sites with a clinical attachment level of at least 5 mm (0.11), and antibiotic duration (0.08). AMX, amoxicillin; BoP, bleeding on probing; CAL, clinical attachment level; MTZ, metronidazole; PPD, probing pocket depth.

Relative feature importance analysis showed collective relative importance's of 0.42 for the 6 clinical, 0.33 for the 3 treatment‐related, 0.21 for the 7 microbiological, and 0.04 for the 2 demographic features. In predicting the clinical endpoint,^34^ the key individual features with the highest relative importance were total daily metronidazole dose (relative feature importance: 0.23), baseline number of sites with PPD ≥ 5 mm (0.17), baseline number of sites with CAL ≥ 5 mm (0.10), and antibiotic treatment duration (0.07). The relative feature importance's of each individual feature are shown in Figure 3.

**FIGURE 4:**
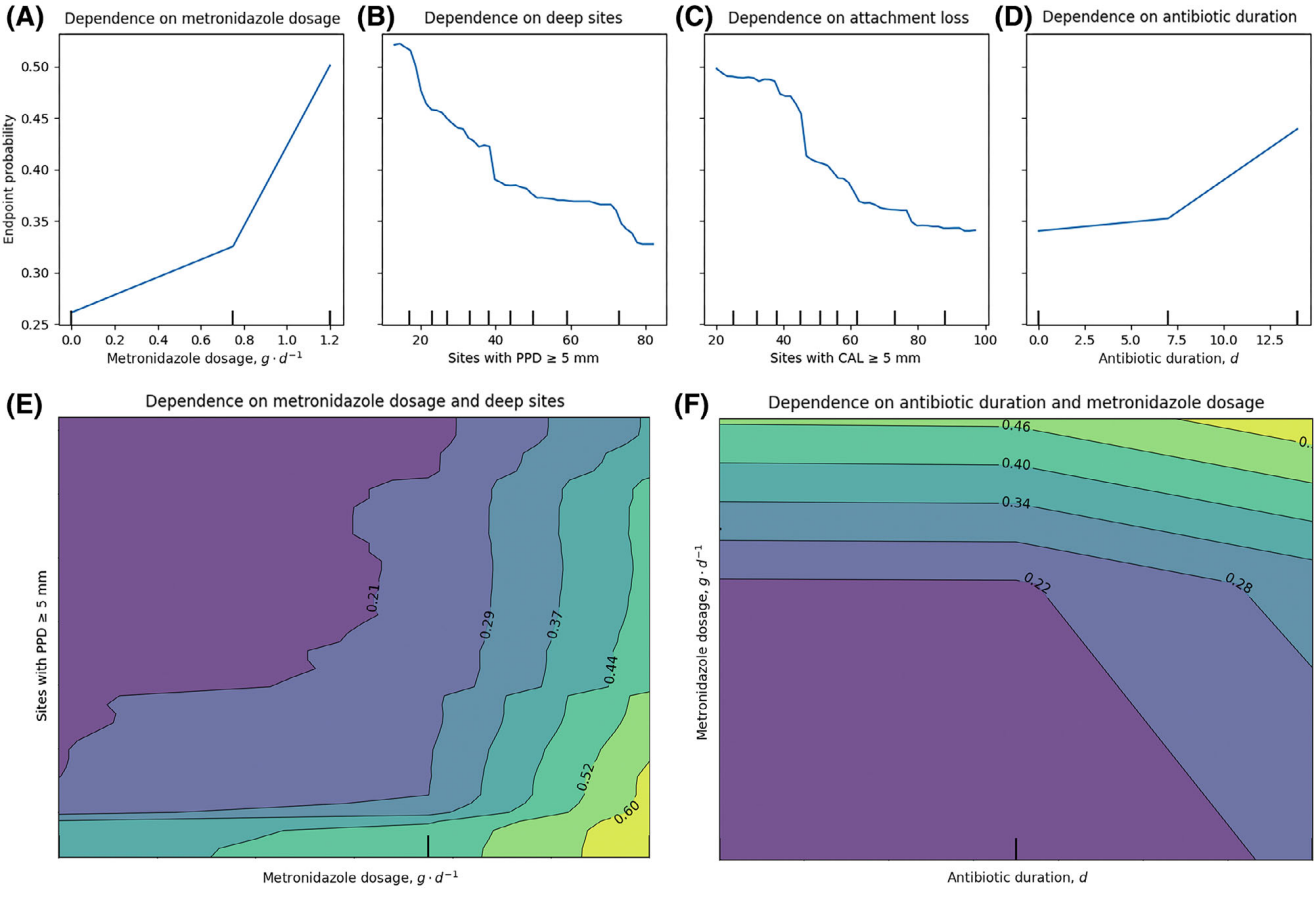
Partial dependence plots. Individual dependence plots are shown for the 4 features with the highest relative importance's, 2‐dimensional dependence plots are shown for clinically relevant interactions. (A, D) Total daily metronidazole dose and antibiotic duration show a positive effect on the model's confidence in achieving the clinical endpoint.^34^ (B, C) Number of sites with a PPD or clinical attachment level of at least 5 mm show a negative effect on the model's confidence in achieving the clinical endpoint.^34^ (E) Interaction effect between the number of sites with PPD ≥ 5 mm and total daily metronidazole dose. (F) Interaction effect between metronidazole dose and antibiotic duration. Ticks represent observed values. CAL, clinical attachment level; PPD, probing pocket depth.

Partial dependence plots for key individual predictive features showed a positive relationship between the achievement of the clinical endpoint^34^ and total daily metronidazole dose as well as antibiotic duration. Of these, daily metronidazole dose showed a more dynamic effect on the prognosis; the predicted probability of the endpoint doubled between the minimal (0.26 for 0 g∙d^−1^) and maximal observed value (0.5 for 1.2 g∙d^−1^) of metronidazole dosage (Figure 4A,D). A negative relationship was observed between the endpoint and the baseline number of sites with PPD ≥ 5 mm or CAL ≥ 5 mm. Both features showed an apparent threshold at approximately 40 sites, where a pronounced drop in the predicted probability of the endpoint was observable (Figure 4, B and C).

A 2‐dimensional partial dependence plot for metronidazole dose and the number of sites with PPD ≥ 5 mm showed a nuanced interaction effect, where the predicted probability of the endpoint increased more sharply when both variables were at higher values. As the number of sites with PPD ≥ 5 mm increased while maintaining a low metronidazole dosage, the predicted probability of the endpoint decreased noticeably. In contrast, at higher levels of metronidazole dosage, the predicted probability of the endpoint stayed relatively stable, even with increasing numbers of sites with PPD ≥ 5 mm (Figure 4E). In the plot for antibiotic duration and metronidazole dose, a non‐linear relationship was observed. The predicted probability of the endpoint remained stable across low to moderate levels of metronidazole dosage as well as most levels of antibiotic duration but increased more sharply beyond an apparent threshold of 0.9 g∙d^−1^ in metronidazole dosage (Figure 4F).

### 3.3 Prognostic performance in external testing

**FIGURE 5:**
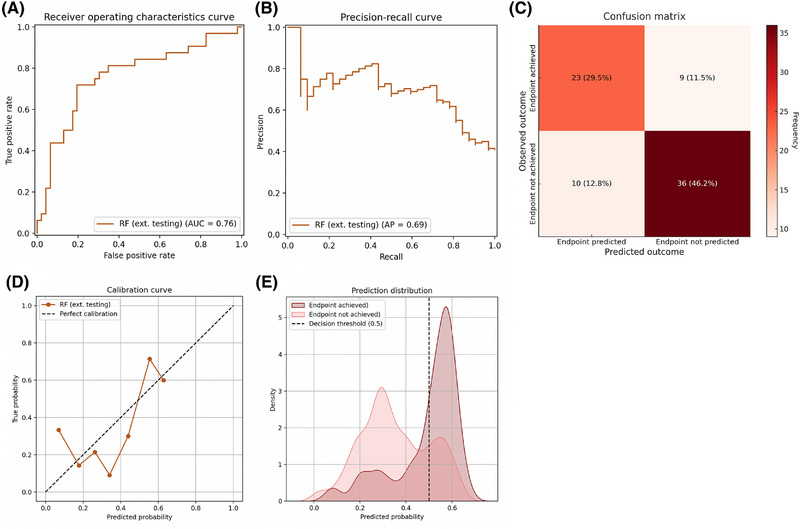
Prognostic model performance in external testing. Performance is shown as evaluated on the North American/European dataset (*n* = 78) not used for training. (A) Receiver operating characteristics curve. (B) Precision‐recall curve. The model achieved a precision of 0.70 and a recall of 0.72. (C) Confusion matrix. The model achieved an accuracy of 0.76. (D) Calibration curve. (E) Prediction distribution. Densities are normalized. AP, average precision; AUC, area under the curve; RF, Random Forest.

In external testing on a secondary dataset of patients from North America and Europe with less severe disease, our prognostic model predicted a mean probability of 41% of achieving the clinical endpoint, compared with an observed events fraction of 41%. The model achieved an AUROC of 0.76 (Figure 5A). The model's precision, recall, and AUPRC were 0.70, 0.72, and 0.69, respectively; the F_1_‐score was 0.71 (Figure 5B). The model achieved an accuracy of 0.76, with an error distribution of 0.12 for false negatives and 0.13 for false positives (Figure 5C). The calibration curve had a slope 0.80 (95% confidence interval: [–0.37, 1.82]; Figure 5D). Distribution analysis revealed slightly weaker separation of predicted outcomes, primarily due to lower predicted probabilities for positive predictions (Figure 5E). Decision curve analysis demonstrated a clear net benefit advantage across relevant threshold probabilities, including the 50% decision threshold (See Figure S1B in online *Journal of Periodontology*). Subgroup analysis based on the number of sites with CAL ≥ 5 mm showed highest model performance in the second quartile (AUROC: 0.78; F_1_‐score: 0.74), followed by the first (AUROC: 0.68; F_1_‐score: 0.87), third (AUROC: 0.78; F_1_‐score: 0.40), and fourth (AUROC: 0.47; F_1_‐score: 0.33) quartiles (See Table S4 in online *Journal of Periodontology*).

### 3.4 Benchmarking

In internal and external testing, respectively, the Extreme Gradient Boosting model achieved an AUROC of 0.96 and 0.72, an AUPRC of 0.94 and 0.64, and an F_1_‐score of 0.88 and 0.52. The Support Vector Machine model achieved an AUROC of 0.82 and 0.65, an AUPRC of 0.73 and 0.60, and an F_1_‐score of 0.70 and 0.50. The K‐Nearest Neighbors model achieved an AUROC of 0.84 and 0.55, an AUPRC of 0.77 and 0.44, and an F_1_‐score of 0.69 and 0.53. Detailed performance comparisons are shown in Table S5 in the online *Journal of Periodontology*.

## 4 DISCUSSION

This study presents the first prognostic model for individual periodontal treatment response trained and tested on well‐defined granular datasets from 8 RCTs conducted in 3 continents—South America, North America, and Europe. We used the Random Forest algorithm on 18 baseline demographic, clinical, microbiological, and treatment‐related features to predict treatment success via the previously validated clinical endpoint.^34^ Internal testing in a South American dataset showed good prognostic performance, most notably an out‐of‐bag score of 0.71 (Figure 2). Interpretability analysis revealed a fairly balanced interplay among clinical disease severity, microbial profiles, and treatment decisions in determining outcomes. Key individual features of high importance, such as the number of deep periodontal pockets and metronidazole dosage, aligned well with clinical plausibility and prior research (Figure 3). External testing on a smaller dataset from 2 different continents, North America and Europe, suggested acceptable generalizability, with an AUROC of 0.76 and an F_1_‐score of 0.71 (Figure 5). Decision curve analysis indicated potential prognostic value (See Figure S1 in online *Journal of Periodontology*), through further validation is required to assess clinical utility. Subgroup analysis based on baseline disease severity showed largely consistent performance, albeit showing a noticeable dip in external testing in the fourth quartile (i.e., highest severity). Compared with other ML algorithms, our Random Forest model performed on par with the Extreme Gradient Boosting model in internal testing and better than the Support Vector Machine and K‐Nearest Neighbors models; in external testing, it outperformed all 3 other algorithms (See Table S5 in online *Journal of Periodontology*). Nonetheless, the limited sample size warrants further validation.

As applied ML gains ground in oral health research, the role of prognostic modeling for risk stratification and individualized prediction is expanding. Recent work has used ML to detect salivary biomarkers for periodontitis from the microbiome^47^ and metabolome.^48^ This increasing application of ML to rich data may enable the identification of complex, implicit patterns within clinical and microbiological data, complementing traditional diagnostic, and prognostic approaches. Our own prognostic ML model was trained on a total of 18 features across demographic, clinical, microbiological, and treatment‐related categories. In the South American dataset used for training, 172 patients reached the endpoint (i.e., event of interest), resulting in an events fraction of approximately 42% and an EPV ratio of 10. In the North American/European dataset used for external testing, 32 patients reached the endpoint, for a comparable events fraction of 41%. As prognostic modeling in dentistry is often complicated by low‐EPV environments (i.e., the number of events of interest is low relative to the number of analyzed predictors), models may become overfit, capturing random patterns and noise in the dataset in addition to, or rather than, real associations. This makes it harder to trust the model's predictions in other populations. Our previous work in very low‐EPV environments (e.g., post‐extraction neuropathy) used regularized regression.^49^, ^50^ In this study, the therapeutic success rate has resulted in a much higher events fraction; we nonetheless consciously engineered patient‐level features from raw retrospective data to maintain an acceptable EPV ratio.

We selected Random Forest as the model architecture for its interpretability, crucial in medical contexts where trust and transparency are essential. Decision tree‐based models offer clear decision pathways, ensuring clinically meaningful and actionable insights. For benchmarking, we compared the Random Forest model's performance against other ML algorithms. In internal testing, the Random Forest and Extreme Gradient Boosting models performed similarly, while the Support Vector Machine and K‐Nearest Neighbors models showed weaker performance. The Random Forest model demonstrated better generalizability in external testing compared to the other 3 algorithms (See Table S4 in online *Journal of Periodontology*).

Relative feature importance analysis showed that our model considers the disease's initial clinical manifestation, the patient's microbial profile, as well as treatment choice as important determinants, with relative importance's ranging from 0.21 to 0.42; experimental omission of any of these 3 feature groups resulted in a considerable reduction in model performance (See Figure S2 in online *Journal of Periodontology*). Among the individual features used to train our ML model, metronidazole dosage and the baseline number of sites with PPD ≥ 5 mm emerged as the most important individual predictors of the clinical endpoint. We further observed a nuanced interaction observed between PPD and metronidazole dosage. While an increased number of sites with PPD ≥ 5 mm generally reduced the likelihood of reaching the clinical endpoint at lower metronidazole doses, this probability remained relatively stable at higher dosages, even as the number of deep sites increased. This finding suggests that higher doses of metronidazole may be associated with improved treatment outcomes, especially in patients with a greater number of deep pockets. Further RCTs directly comparing different drug dosages are needed to confirm this observation. Additionally, expanding the model's training with datasets incorporating varied antibiotic doses could further enhance its predictive accuracy regarding dosing strategies. An unexpected result was the relatively low importance of amoxicillin for model predictions. Mechanical therapy combined with amoxicillin and metronidazole is currently widely regarded as the most effective treatment for severe periodontitis, as demonstrated by numerous clinical trials and systematic reviews.^41^, ^51^, ^52^ These findings are particularly noteworthy in the broader context of growing concerns about antibiotic resistance. Such insights not only help in refine treatment strategies but also support efforts to avoid overprescription, ultimately minimizing unnecessary drug use and reducing treatment costs.

The main strength of our study lies in the dataset quality. To our knowledge, our datasets of raw, site‐level measurements by calibrated researchers from 8 RCTs, are among the most comprehensive and granular datasets of periodontal patients to date; they have enabled us to externally test our model on patients across multiple continents. Further strengths include a comprehensive interpretability analysis, often absent in similar work. Additionally, we benchmarked our results against 3 mainstream ML architectures.

Limitations of our model include the small dataset size which constrained model robustness. Our algorithm further considers data as independent and identically distributed (iid); however, retrospective data collection from multiple centers likely disrupted the true iid nature of the data. Additionally, feature assessment was not blinded during model development and testing, and the engineering of patient‐level features from raw, site‐level data introduces a loss of granularity, including spatial disease distribution. This could be addressed by employing a graph neural network to capture potential spatial dependencies. A further limitation is that we only use 1 time point (baseline) to predict outcomes at 1 time point (1 year post‐treatment). Leveraging multiple time points (e.g., by using a recurrent neural network) could potentially improve predictions by incorporating longitudinal information on the disease resolution trajectory as well as patient adherence. We note that we already utilize all available pre‐treatment information; by the nature of periodontal treatment, additional time points would be post‐treatment. A limitation to external testing is retrospective data collection; more robust results could be obtained by adopting a prospective framework. Finally, we only included patients with complete data, assessing completeness at the tooth level. While we could exclude patients with clearly incomplete records (e.g., missing individual sites), we assumed that if all sites for a tooth were missing, the tooth had likely been lost rather than representing missing data. This may have introduced bias by including patients whose teeth lacked recordings for all 6 sites.

The clinical relevance of prognostic models incorporating treatment‐related features lie in their potential to individualize periodontal treatment decision‐making. Currently, no validated instrument exists to predict individual treatment response, requiring clinicians and patients to assume that strict adherence to treatment guidelines will lead to favorable outcomes. However, the variability in patient responses to different treatment modalities highlights the need for personalized approaches. This is supported by our decision curve analysis, demonstrating a clear advantage of our prognostic model over the assumption that all patients will achieve the clinical endpoint following treatment.

The use of commonly collected clinical features may facilitate future integration of this or similar prognostic models into clinical workflows through clinician‐facing software, though usability and implementation studies are needed. By evaluating the baseline condition and generating predictions for various treatment options (e.g., different active agents, dosages), clinicians can select the least intensive adjunctive treatment that still ensures the patient is likely to reach the clinical endpoint. Importantly, this would help limit drug overuse while ensuring treatment efficacy.

The clinical implementation of ML models poses several challenges. A previous analysis of diagnostic and prognostic models for coronavirus disease 2019 has concluded poor reporting and high risk of bias, limiting clinical utility.^53^ While we already focus on geographical diversity, future iterations of our model could reduce bias by incorporating even more patient populations, data types (e.g., patient‐reported outcomes, host immunoinflammatory features), and treatment modalities (e.g., host modulation therapy, reconstructive surgery). A broader range of clinical scenarios would further enable to model to provide a more comprehensive foundation for decision‐making.

From a global health perspective, enhancing the predictability of periodontal treatment is essential, as the disease not only compromises oral health but also has profound systemic consequences.^54^, ^55^, ^56^ Considering the rising global prevalence of periodontitis and the absence of personalized treatment approaches, models like ours have the potential to benefit a large number of patients worldwide. These approaches might pave the way for more targeted clinical care and may help reduce the broader health and economic burdens associated with this widespread condition.

In conclusion, our study indicates that an ML‐based approach can assist in predicting individual responses to periodontal treatment. Prospective validation is needed for clinical application.

## AUTHOR CONTRIBUTIONS

Balazs Feher contributed to conceptualization, data curation, formal analysis, investigation, methodology, validation, and visualization, and drafted the manuscript. Eduardo H. de Souza Oliveira contributed to conceptualization, data curation, methodology, and resources, and critically revised the manuscript. Andreas A. Werdich contributed to formal analysis and methodology and critically revised the manuscript. Poliana Duarte contributed to conceptualization, data interpretation and critically revised the manuscript. William V. Giannobile contributed to data curation, investigation, visualization, and critically revised the manuscript. Magda Feres contributed to conceptualization, methodology, project administration and supervision, and critically revised the manuscript. All authors gave their final approval and agreed to be accountable for all aspects of the work.

## CONFLICT OF INTEREST STATEMENT

The authors have no conflicts of interest, financial or otherwise, to disclose.

## INSTITUTIONAL REVIEW BOARD STATEMENT

As a secondary analysis of existing, deidentified data, institutional review board approval was deemed exempt. The protocols of the individual RCTs used for data collection were approved by the respective institutional review boards: Guarulhos University Clinical Research Ethics Committee (protocol Nos. SISNEP/513,^35^ SISNEP/697,^36^ SISNEP/229,^37^ SISNEP/225,^38^ SISNEP/726); Federal University of Rio de Janeiro Ethics Committee (protocol No. 47‐00^39^); Boston University Medical Campus Institutional Review Board (protocol No. 5147^43^); and Forsyth Institute Institutional Review Board (protocol No. 00‐02^44^). Four trials were further registered at ClinicalTrials.gov (protocol Nos.: NCT02735395,^35^ NCT02135952,^36^ NCT00127244,^43^ NCT06177119).

## Supporting information



Supporting Information

Supporting Information

## Data Availability

A synthetic dataset generated from the original data as well as the underlying code for this study are available in our repository and can be accessed via this link: https://github.com/balazsfeher/perio‐prediction. The original dataset is unavailable as it consists of secondary data from multiple clinical studies without patient consent for public disclosure of raw data.
