# Supplementary material for: Machine learning‐assisted prediction of clinical responses to periodontal treatment
Source: J Periodontol. 2025 Apr 20;96(11):1199–212. doi: 10.1002/JPER.24-0737 (PMC12671691; doi:10.1002/JPER.24-0737)
Supplement: Supplementary file 1 — Supporting Information [file JPER-96-1199-s002.pdf]

Predicting individual responses to periodontal treatment using machine learning

Balazs Feher, Eduardo H. de Souza Oliveira, Poliana Duarte, Andreas A. Werdich, William V. Giannobile, Magda Feres

Supplementary Figures

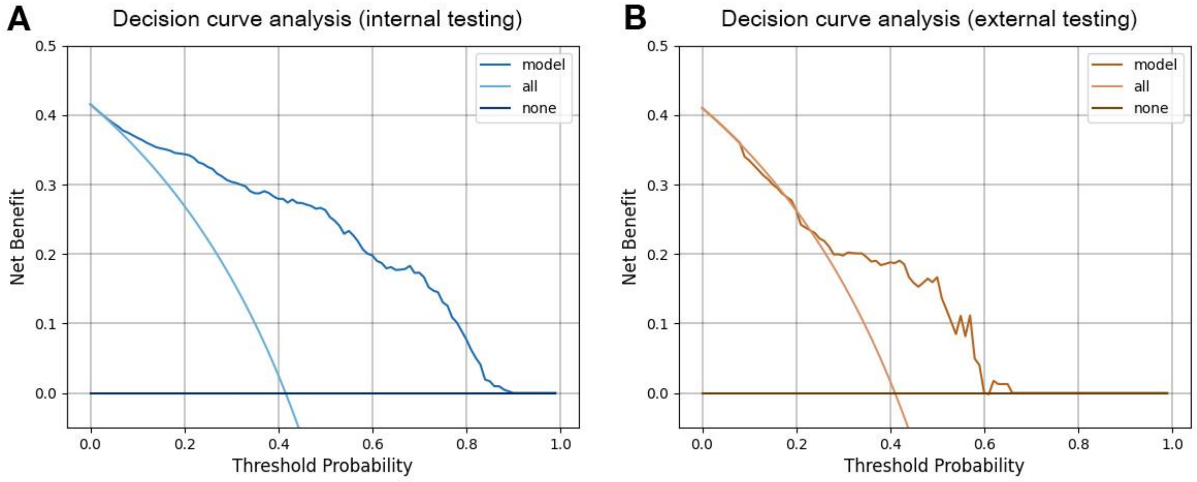

Supplementary Figure 1: Decision curve analysis shows clinical utility of the prognostic model. In both the **A** South American dataset used for training and the **B** North American/European dataset not used for training**,** decision curve analysis demonstrated the clinical utility of the prognostic model compared to the “all” (i.e., treatment inevitably leads to achievement of the endpoint) and “none” (i.e., no patient achieves the endpoint) approaches, showing a net benefit across relevant threshold probabilities, including a clear advantage at the chosen 50% threshold**.**

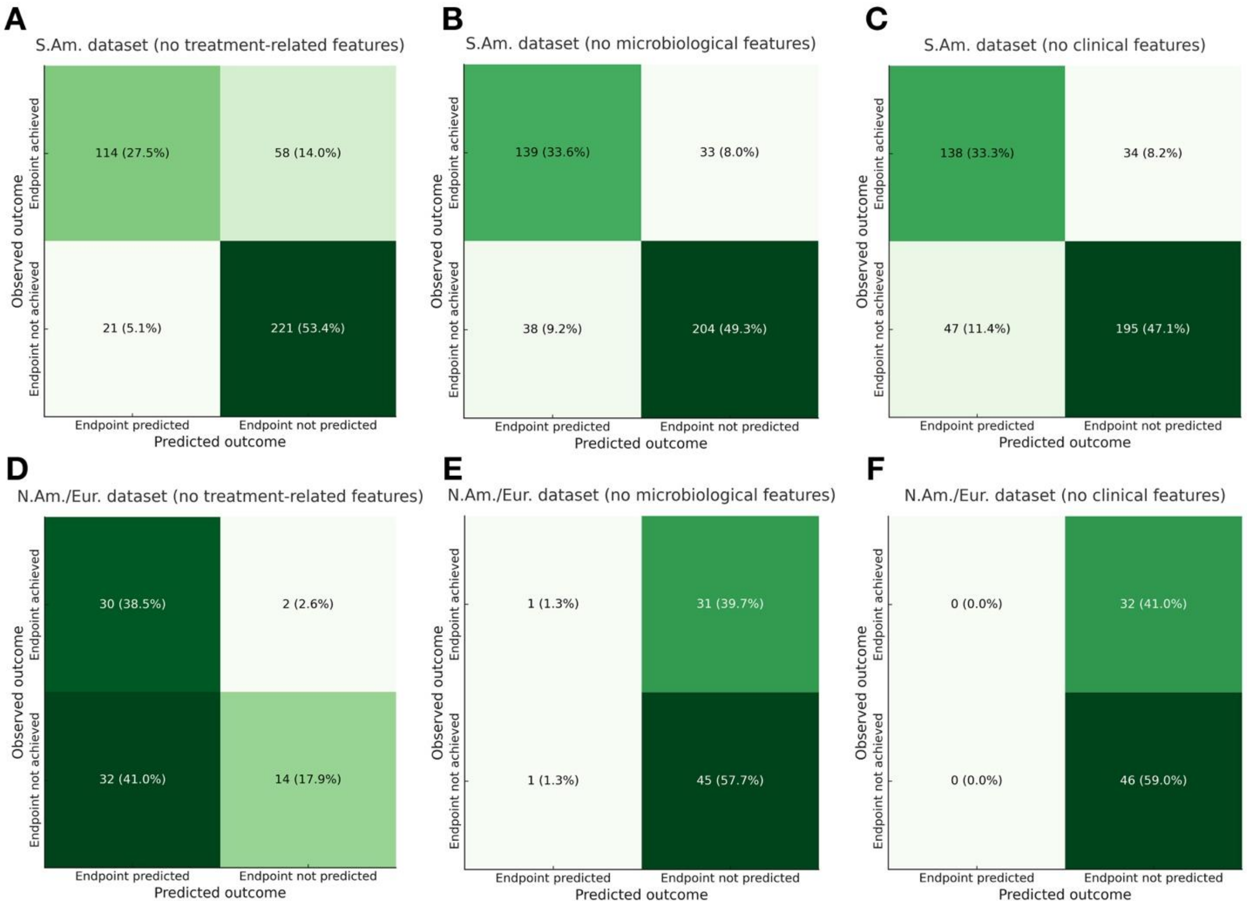

Supplementary Figure 2: Experimental omission of feature categories reduces generalizability. (A–C) In the South American dataset used for training, where the complete model achieved an accuracy of 0.85, omitting feature categories did not meaningfully impact model performance. Omitting treatment-related features reduced accuracy to 0.81; omitting microbiological features reduced accuracy to 0.83; omitting clinical features reduced accuracy to 0.80. **(D–F)** In the North American/European dataset not used for training, where the complete model achieved an accuracy of 0.76, omitting feature categories drastically reduced model performance. Omitting treatment-related features reduced accuracy to 0.56; omitting microbiological or clinical features each reduced accuracy to 0.59.
