# Supplementary material for: Machine learning‐assisted prediction of clinical responses to periodontal treatment
Source: J Periodontol. 2025 Apr 20;96(11):1199–212. doi: 10.1002/JPER.24-0737 (PMC12671691; doi:10.1002/JPER.24-0737)
Supplement: Supplementary file 2 — Supporting Information [file JPER-96-1199-s001.docx]

**Predicting individual responses to periodontal treatment using machine learning**

Balazs Feher, Eduardo H. de Souza Oliveira, Poliana Duarte, Andreas A. Werdich, William V. Giannobile, Magda Feres

**Supplementary Tables**

**Supplementary Table 1: Data sources.**

| **Trial** | **Dataset allocation** | **Number of patients** | **Age (median, years)** | **Sex** | **Events fraction** | **Data collection**  **(year-month)** | |
| --- | --- | --- | --- | --- | --- | --- | --- |
|  |  |  |  |  |  | **Start** | **End** |
| Carvalho et al. (2004),^1^  Carvalho et al. (2005)^2^ | S.Am. | 38 | 43 | 61% female | 21% | 2000-05 | 2003-12 |
| Mestnik et al. (2012)^3^ | S.Am. | 30 | 27 | 57% female | 23% | 2007-07 | 2010-12 |
| Feres et al. (2012),^4^  Soares et al. (2014)^5^ | S.Am. | 118 | 44 | 82% female | 50% | 2009-02 | 2011-08 |
| Miranda et al. (2014)^6^,  Tamashiro et al. (2016)^7^ | S.Am. | 52 | 55 | 44% female | 46% | 2011-09 | 2012-10 |
| Borges et al. (2017)^8^ | S.Am. | 109 | 47 | 57% female | 33% | 2011-07 | 2014-05 |
| Fermiano et al. (2024, unpublished) | S.Am. | 67 | 42 | 61% female | 57% | 2012-01 | 2013-07 |
| Haffajee et al. (2007)^9^ | N.Am./Eur. | 33 | 44 | 30% female | 61% | 2004-02 | 2006-2 |
| Goodson et al. (2012)^10^ | N.Am./Eur. | 45 | 50 | 38% female | 27% | 1999-03 | 2004-01 |

Eur., Europe; N.Am., North America; S.Am., South America.**Supplementary Table 2: Benchmarking model hyperparameters.**

| **Hyperparameter** | **Optimal value** |
| --- | --- |
| Extreme Gradient Boosting | |
| Number of gradient-boosted trees | 100 |
| Maximum tree depth | 10 |
| Learning rate | 0.05 |
| Training subsample ratio | 0.7 |
| Column sample ratio | 0.7 |
| L1 penalization term | 1.5 |
| L2 penalization term | 10.5 |
| Loss reduction threshold | 1 |
| Support Vector Machine | |
| Kernel | Linear |
| Regularization parameter | 5 |
| Kernel coefficient | 1.5 |
| K-Nearest Neighbors | |
| Number of neighbors | 9 |
| Weighting scheme | Uniform |
| Distance metric | Euclidean |

**Supplementary Table 3: Subgroup analysis of the Random Forest prognostic model in internal testing.**

| **Number of sites with  CAL ≥ 5 mm** | **Subgroup size** | **AUROC** | **AUPRC** | **Precision** | **Recall** | **F_1_-score** |
| --- | --- | --- | --- | --- | --- | --- |
| 1^st^ quartile (11 ≤ n < 35) | 107 | 0.91 | 0.95 | 0.80 | 0.89 | 0.84 |
| 2^nd^ quartile (35 ≤ n < 51) | 101 | 0.93 | 0.93 | 0.83 | 0.85 | 0.84 |
| 3^rd^ quartile (51 ≤ n < 68) | 105 | 0.94 | 0.90 | 0.86 | 0.81 | 0.83 |
| 4^th^ quartile (68 ≤ n < 134) | 101 | 0.92 | 0.78 | 0.78 | 0.56 | 0.65 |

AUPRC, area under the precision-recall curve; AUROC, area under the receiver operating characteristics curve; CAL, clinical attachment loss.

**Supplementary Table 4: Subgroup analysis of the Random Forest prognostic model in external testing.**

| **Number of sites with  CAL ≥ 5 mm** | **Subgroup size** | **AUROC** | **AUPRC** | **Precision** | **Recall** | **F_1_-score** |
| --- | --- | --- | --- | --- | --- | --- |
| 1^st^ quartile (0 ≤ n < 13) | 20 | 0.68 | 0.77 | 0.81 | 0.93 | 0.87 |
| 2^nd^ quartile (13 ≤ n < 25) | 19 | 0.78 | 0.83 | 0.78 | 0.70 | 0.74 |
| 3^rd^ quartile (25 ≤ n < 45) | 19 | 0.78 | 0.61 | 0.33 | 0.50 | 0.40 |
| 4^th^ quartile (45 ≤ n < 111) | 20 | 0.47 | 0.30 | 0.50 | 0.25 | 0.33 |

AUPRC, area under the precision-recall curve; AUROC, area under the receiver operating characteristics curve; CAL, clinical attachment loss.

**Supplementary Table 5: Performance comparison of various machine learning algorithms.**

| **Model** | **AUROC** | | **Accuracy** | | **AUPRC** | | **Precision** | | **Recall** | | **F_1_-score** | |
| --- | --- | --- | --- | --- | --- | --- | --- | --- | --- | --- | --- | --- |
|  | **Int.** | **Ext.** | **Int.** | **Ext.** | **Int.** | **Ext.** | **Int.** | **Ext.** | **Int.** | **Ext.** | **Int.** | **Ext.** |
| Random Forest | 0.93 | 0.76 | 0.85 | 0.76 | 0.90 | 0.69 | 0.82 | 0.70 | 0.81 | 0.72 | 0.82 | 0.71 |
| Extreme Gradient Boosting | 0.96 | 0.72 | 0.94 | 0.64 | 0.92 | 0.58 | 0.88 | 0.58 | 0.88 | 0.47 | 0.88 | 0.52 |
| Support Vector Machine | 0.82 | 0.65 | 0.74 | 0.63 | 0.73 | 0.60 | 0.67 | 0.56 | 0.72 | 0.47 | 0.70 | 0.50 |
| K-Nearest Neighbors | 0.84 | 0.55 | 0.73 | 0.56 | 0.77 | 0.44 | 0.67 | 0.48 | 0.70 | 0.59 | 0.69 | 0.53 |

AUPRC, area under the precision-recall curve; AUROC, area under the receiver operating characteristics curve.

**References**

1. Carvalho LH, D'Avila GB, Leão A, Haffajee AD, Socransky SS, Feres M. Scaling and root planing, systemic metronidazole and professional plaque removal in the treatment of chronic periodontitis in a Brazilian population. I. clinical results. *J Clin Periodontol*. Dec 2004;31(12):1070-6. doi:10.1111/j.1600-051X.2004.00605.x

2. Carvalho LH, D'Avila GB, Leão A, et al. Scaling and root planing, systemic metronidazole and professional plaque removal in the treatment of chronic periodontitis in a Brazilian population II--microbiological results. *J Clin Periodontol*. Apr 2005;32(4):406-11. doi:10.1111/j.1600-051X.2005.00720.x

3. Mestnik MJ, Feres M, Figueiredo LC, et al. The effects of adjunctive metronidazole plus amoxicillin in the treatment of generalized aggressive periodontitis: a 1-year double-blinded, placebo-controlled, randomized clinical trial. *J Clin Periodontol*. Oct 2012;39(10):955-61. doi:10.1111/j.1600-051X.2012.01932.x

4. Feres M, Soares GM, Mendes JA, et al. Metronidazole alone or with amoxicillin as adjuncts to non-surgical treatment of chronic periodontitis: a 1-year double-blinded, placebo-controlled, randomized clinical trial. *J Clin Periodontol*. Dec 2012;39(12):1149-58. doi:10.1111/jcpe.12004

5. Soares GM, Mendes JA, Silva MP, et al. Metronidazole alone or with amoxicillin as adjuncts to non-surgical treatment of chronic periodontitis: a secondary analysis of microbiological results from a randomized clinical trial. *J Clin Periodontol*. Apr 2014;41(4):366-76. doi:10.1111/jcpe.12217

6. Miranda TS, Feres M, Perez-Chaparro PJ, et al. Metronidazole and amoxicillin as adjuncts to scaling and root planing for the treatment of type 2 diabetic subjects with periodontitis: 1-year outcomes of a randomized placebo-controlled clinical trial. *J Clin Periodontol*. Sep 2014;41(9):890-9. doi:10.1111/jcpe.12282

7. Tamashiro NS, Duarte PM, Miranda TS, et al. Amoxicillin Plus Metronidazole Therapy for Patients with Periodontitis and Type 2 Diabetes: A 2-year Randomized Controlled Trial. *J Dent Res*. Jul 2016;95(7):829-36. doi:10.1177/0022034516639274

8. Borges I, Faveri M, Figueiredo LC, et al. Different antibiotic protocols in the treatment of severe chronic periodontitis: A 1-year randomized trial. *J Clin Periodontol*. Aug 2017;44(8):822-832. doi:10.1111/jcpe.12721

9. Haffajee AD, Torresyap G, Socransky SS. Clinical changes following four different periodontal therapies for the treatment of chronic periodontitis: 1-year results. *J Clin Periodontol*. Mar 2007;34(3):243-53. doi:10.1111/j.1600-051X.2006.01040.x

10. Goodson JM, Haffajee AD, Socransky SS, et al. Control of periodontal infections: a randomized controlled trial I. The primary outcome attachment gain and pocket depth reduction at treated sites. *J Clin Periodontol*. Jun 2012;39(6):526-36. doi:10.1111/j.1600-051X.2012.01870.x
